# T-cell responses to primary SARS-CoV-2 vaccination in Down syndrome – From childhood to adulthood

**DOI:** 10.1080/21645515.2026.2670839

**Published:** 2026-06-01

**Authors:** Lobke C. M. Hensen, Bianca M. M. Streng, Femke van Wijk, Stefan Nierkens, Joanne G. Wildenbeest, Louis J. Bont, Eveline M. Delemarre, the PRIDE study group

**Affiliations:** a Department of Pediatric Infectious Diseases and Immunology, Wilhelmina Children’s Hospital, University Medical Center Utrecht, Utrecht, The Netherlands; b Center for Translational Immunology, University Medical Center Utrecht, Utrecht University, Utrecht, The Netherlands; c Princess Máxima Center for Pediatric Oncology, Utrecht, The Netherlands; Laboratory for Vaccine-Preventable Diseases, National Institute of Public Health and the Environment, Bilthoven, The Netherlands; Primary and Community Care, Radboud University Medical Center, Nijmegen, The Netherlands; Viroscience, Erasmus University Medical Center, Rotterdam, The Netherlands; foundation ‘Stichting Downsyndroom’, Meppel, The Netherlands; Sanquin Research and Landsteiner Laboratory, Amsterdam University Medical Center, University of Amsterdam, Amsterdam, The Netherlands; Biomolecular Mass Spectrometry and Proteomics, Utrecht Institute for Pharmaceutical Sciences and Bijvoet Center for Biomolecular Research, Utrecht University, Utrecht, The Netherlands; Vaccine Research Center, National Institute of Allergy and Infectious Disease, Bethesda, Maryland, USA; Tranzo, Tilburg School of Social and Behavioral Sciences, Tilburg University, Tilburg, The Netherlands; Academy Research, Jeroen Bosch Hospital, ‘s-Hertogenbosch, The Netherlands; Pediatrics, Alrijne Hospital, Leiderdorp, The Netherlands; Epidemiology of Microbial Diseases, Yale School of Public Health, New Haven, Connecticut, USA

**Keywords:** Children, Down syndrome, COVID-19, SARS-CoV-2, mRNA vaccine, T cells, IFNγ

## Abstract

Down syndrome (DS) is the most common genetic disorder worldwide and associated with high morbidity and mortality rates during the COVID-19 pandemic. For safety reasons, SARS-CoV-2 vaccine schedules and dosages were age-dependent, with children <12 years (y) receiving a lower dose than adolescents and adults. While we previously reported age-dependent antibody responses after SARS-CoV-2 vaccination in children with DS, cellular vaccine responses in this population remain insufficiently characterized. We evaluated vaccine-induced T-cell responses in children with DS following primary mRNA SARS-CoV-2 vaccination. We measured SARS-CoV-2-specific T-cell abundance (N = 40) and their interferon-gamma (IFNγ) production (N = 55) after spike antigen re-stimulation in participants aged 3–74 y. We found no significant difference in the re-activation of SARS-CoV-2-specific CD4^+^ T cells between adolescents (12–17 y) and adults. Children aged 5–11 y exhibited significantly lower re-activation of CD4^+^ T cells compared with adolescents. IFNγ production was similar across all age groups. Besides previously reported age-dependent antibody responses, these findings suggest that reduced dosing may also be associated with diminished T-cell re-activation in children with DS. Therefore, these children aged 5–11 y may benefit from receiving a booster or vaccine doses similar to those of older age groups to ensure optimal immunity and protection.

**Clinical trial registration**: NCT05145348.

Down syndrome (DS) is caused by a triplication of chromosome 21 and is the most prevalent genetic disorder worldwide, with an incidence of 9.9/10,000 livebirths in the Netherlands.^1^ Individuals with DS present with a variety of co-occurring conditions such as intellectual disability to a varying degree,^2^ congenital heart disease,^3^ and immune dysregulation.^4–9^ Besides non-immunologic features such as structural airway abnormalities and low muscle tone, immune dysregulation within the innate and adaptive immune compartments is thought to underlie the increased risk of severe respiratory infections observed in individuals with DS.^5,10–12^ During the coronavirus disease 2019 (COVID-19) pandemic, adults with DS displayed higher hospitalization and mortality rates compared to the general population, with up to 3–10-fold increased risk of death upon a severe acute respiratory syndrome coronavirus 2 (SARS-CoV-2) infection.^13,14^ Age was an important risk factor; hospitalized adults with DS were younger and experienced more severe illness compared to people without DS,^15^ with mortality rates increasing even further in DS from age 40.^14^ In high-income countries, children with DS had more symptomatic disease resulting in hospitalization, but mortality rates did not increase.^16,17^ However, in a middle-income country (Brazil) children with DS had a 1.8 times higher odds of dying from a SARS-CoV-2 infection than children without DS.^18^ This shows the need for an effective vaccine in this vulnerable population.

SARS-CoV-2 vaccination has proven to be safe and protective against mortality and severe disease in adults with DS.^19,20^ Despite vaccination being safe, DS remains a risk factor for severe disease upon infection, even after two or three vaccine doses.^21^ The efficacy of SARS-CoV-2 vaccination has not been studied in children with DS. For adults with DS, it has been shown that antibody responses were lower after both primary and booster vaccinations compared with controls,^22–25^ with decreasing titers in older adults.^22,25^ On a cellular level, mRNA vaccination induced lower percentages of SARS-CoV-2-specific CD4^+^ T cells in adults with DS, although SARS-CoV-2-specific T cells were functionally as effective compared with healthy controls (HC).^26^ During the pandemic, mRNA vaccination was recommended in the Netherlands for children aged 5–17 y.^27,28^ For children aged 5–11 y, this recommendation was later restricted to those at increased medical risk.^29^ For the youngest age group of children between 6 months and 6 y, the recommendation for vaccination with an mRNA vaccine was limited to those with a high risk of severe COVID-19,^30^ including children with DS. Children received vaccination dosages and schedules dependent on their age.^31^ The primary vaccination in children with DS induced age-specific antibody responses, with lower titers in children below 12 y of age compared to adolescents aged 12–17 y.^25^ The cellular vaccine-induced immune response in children with DS remains poorly understood and has not been studied before for SARS-CoV-2 vaccination, and only to a limited extent for other childhood vaccines.^32^ Here, we evaluated the SARS-CoV-2 vaccine-induced T-cell response in children with DS after primary mRNA vaccination in the context of the PRIDE study.

The PRIDE study is a prospective, observational cohort study in the Netherlands set up to evaluate immune responses in DS and HC following SARS-CoV-2 vaccination, approved by the University Medical Center Utrecht medical research ethics committee (NL76336.041.21).^22,26^ All participants and/or legal representatives provided written informed consent before inclusion. Participants in the PRIDE study were vaccinated according to the Dutch national immunization program, with age-specific vaccine schedules and dosages^31^ (Table 1). To study the recall responses of T-cells after primary vaccination over age, we included all pediatric participants with DS from the PRIDE study as well as adults vaccinated with mRNA vaccinations, either Pfizer or Moderna, with available samples drawn approximately 28 days (d) after completing the primary vaccination schedule (Figure 1). Pediatric HC samples were not available for cellular analyses, as children typically experienced a mild disease course after infection and were therefore not vaccinated and thus not enrolled in the PRIDE study. Additionally, vaccination in the younger children below 12 y of age was restricted to those at increased risk of severe disease, further limiting the possibility to include them in the study.^29,30^

**Figure 1.**
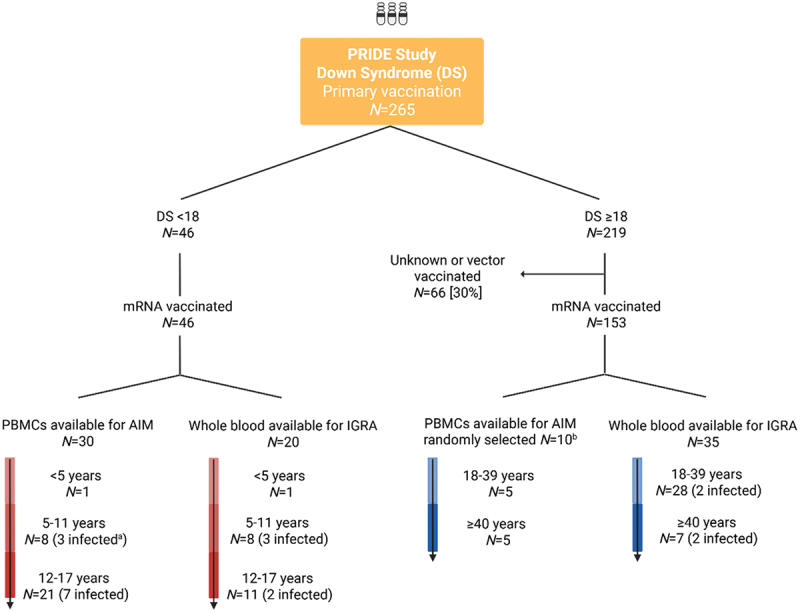
Flow diagram of included Down syndrome (DS) participants from the PRIDE study after primary mRNA vaccination. Number of included participants for which the vaccine-induced T-cell response was investigated with the activation induced marker (aim) assay of peripheral blood mononuclear cells (PBMCs) and with the interferon gamma release assay (IGRA) performed on whole blood. Blood samples for the aim assay and IGRA were all drawn approximately 28 d after completing the primary vaccination schedule. This vaccine was directed against wild-type SARS-CoV-2.

^a^Previous infection with SARS-CoV-2 was determined based on a positive anti-S IgG concentration (>10.08 BAU/ml) at baseline or a positive anti-N IgG concentration (>14.3 BAU/ml) at any time during baseline and sample collection after primary vaccination (baseline (before start primary vaccinations), T2 (approximately 28 d after first vaccination) and T3 (approximately 28 d after second vaccination)^33^.

^b^We randomly selected 10 uninfected adult participants, 5 < 40 and 5 ≥ 40 y of age, from available PBMC samples after primary vaccination.

Created in BioRender. Hensen, L. (2026) https://BioRender.com/kh9mmiw.

**Table 1.** Primary mRNA vaccination schedules and dosages in the PRIDE study according to the Dutch national immunization program.

Age group (years)	Vaccine	Brand	Number of vaccines	Dosage mRNA per injection	Vaccine interval
<5	Comirnaty (Omicron XBB.1.5)	Pfizer/BioNTech	3	3 µg in 0.3 mL	1st–2nd : 3 weeks 2nd–3rd : 8 weeks
5–11	Comirnaty (BNT162b2)	Pfizer/BioNTech	2	10 µg in 0.3 mL	4–8 weeks
12–17	Comirnaty (BNT162b2)	Pfizer/BioNTech	2	30 µg in 0.3 mL	3 weeks
≥18	Comirnaty (BNT162b2)	Pfizer/BioNTech	2	30 µg in 0.3 mL	3–6 weeks
	Moderna (mRNA1273)	Moderna	2	100 µg in 0.5 mL	4–6 weeks

We investigated the cellular vaccine-induced response by measuring the percentage of activated SARS-CoV-2-specific CD4^+^ and CD8^+^ T cells after re-stimulation with spike peptides using the activation induced marker (AIM) assay^26^ (Supplemental Figure 1 and Supplemental Table 1). The AIM assay detects up-regulation of antigen-specific T cell activation markers after re-stimulation. Furthermore, we defined the induction (stimulation index (SI) of SARS-CoV-2-specific T cells (AIM^+^ cells) after S-peptide stimulation using the formula $\mathrm{SI}=\frac{\%\mathrm{AIM}\;+\;\mathrm{cells}\;\mathrm{after}\;S\;-\;\mathrm{peptide}\;\mathrm{stim}}{\%\mathrm{AIM}\;+\;\mathrm{cells}\;\mathrm{in}\;\mathrm{DMSO}\;\left(\mathrm{background}\right)}$. In short, peripheral blood mononuclear cells (PBMCs) were re-stimulated with a wild-type SARS-CoV-2 spike peptide pool consisting of 315 peptides with 15-mer length.^35^ PBMCs were stimulated with an equivalent amount of dimethyl sulfoxide (DMSO, background) to set cutoffs for expression of activation markers, and with αCD3/αCD28 dynabeads as positive control. To identify SARS-CoV-2-specific T cells with flow cytometry in the AIM assay, first memory T cells were defined as CD45RO^+^ within the CD4^+^ and CD8^+^ T-cell subsets. Hereafter, SARS-CoV-2-specific memory CD4^+^ T cells (AIM^+^ CD4^+^ T cells) are defined as CD134^+^CD137^+^ and SARS-CoV-2-specific memory CD8^+^ T cells as CD69^+^CD137^+^ (AIM^+^ CD8^+^ T cells, Supplemental Figure 1).^34^ Additionally, we quantified interferon gamma (IFNγ) production by SARS-CoV-2-specific T cells with the IFNγ release assay (IGRA) as performed before,^26^ using the commercial Quan-T-Cell SARS-CoV-2 kit (EUROIMMUN) and IFNγ ELISA kit Quan-T-Cell ELISA (EUROIMMUN) (Supplemental Table 1) according to the manufacturer’s protocol.

With αCD3/αCD28 dynabead stimulation, we observed significantly increased activation of both CD4^+^ and CD8^+^ T cells in children with DS aged 5–11 y compared to adults (CD4^+^CD134^+^CD137^+^ *p* = .019, CD8^+^CD69^+^CD137^+^ *p* = .0102, data not shown), but no difference between adults, and adolescents. This indicates that T cells in children and adolescents with DS are not impaired in their capacity to become activated and may even show enhanced activation capacity compared to adults with DS.

We found no difference in the SI of SARS-CoV-2-specific CD4^+^ T cells after re-activation of the PBMCs with S-peptides between adolescents aged 12–17 y and adults with DS (Figure 2) after primary vaccination. However, children between 5–11 y showed a significant lower SI of AIM^+^CD4^+^ T cells compared to adolescents. These results held true when looking at the percentage of re-activated SARS-CoV-2-specific CD4^+^ T cells after S-peptide stimulation after background subtraction (Supplemental Figure 2). We found no correlation between age and the SI of SARS-CoV-2-specific CD4^+^ T cells (Spearman’s rank correlation r = 0.2667, *p* = .0962, data not shown).

**Figure 2.**
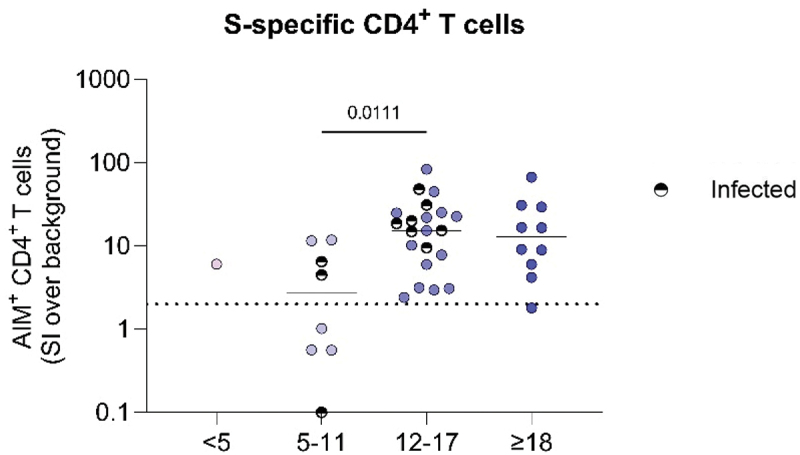
Lower stimulation index of SARS-CoV-2-specific CD4^+^ T cells in children with DS between 5–11 y of age after mRNA primary vaccination. Stimulation index (SI) of AIM^+^CD4^+^ T cells, calculated by dividing specific activation (% AIM^+^ cells after S-peptide stimulation) over background activation (% AIM^+^ cells after DMSO stimulation (background)). AIM^+^CD4^+^ cells are defined as CD134^+^CD137^+^. An SI of 2 or higher is considered a positive T-cell response (dashed line). Significance was determined using Kruskal-Wallis test with Dunn’s multiple comparisons test. The single participant <5 was not taken along for calculating significance. The median is shown as a black line. Previous infected individuals were annotated as infected based on criteria as described in Figure 1. DS < 5 N = 1, DS 5–11 N = 8, DS 12–17 N = 21, DS ≥ 18 N = 10.

For CD8^+^ SARS-CoV-2-specific T cells, we found a decreased SI in the age group 5–11 y compared to adolescents (Supplemental Figure 3(A)), although this difference was not observed in the percentage of re-activated CD8^+^ SARS-CoV-2-specific T cells (Supplemental Figure 3(B)). We observed a significant higher percentage of re-activated SARS-CoV-2-specific CD8^+^ T cells in adolescents compared to adults.

We did not exclude infected children and adolescents for our initial analysis due to limited sample availability, but performed a sensitivity analysis without previous infected individuals. After exclusion, we still observed a significant lower percentage of re-activated SARS-CoV-2-specific CD4^+^ T cells in children aged 5–11 y compared to adolescents (*p* = .0325). We did not find a difference in the SI of both CD4^+^ and CD8^+^ SARS-CoV-2-specific T cells between any of the age groups. The timing of when individuals got infected can be found in Supplemental Table 2.

To investigate the functional T-cell re-activation response, we quantified IFNγ production by memory SARS-CoV-2-specific T cells (CD4^+^ and CD8^+^ combined). We observed no difference in the amount of IFNγ produced after primary vaccination between the different age groups (Figure 3). Additionally, there was no correlation between IFNγ production and age (Spearman’s rank correlation r = −0.08, *p* = .5551, data not shown). The timing of a previous infection for each participant is listed in Supplemental Table 3.

**Figure 3.**
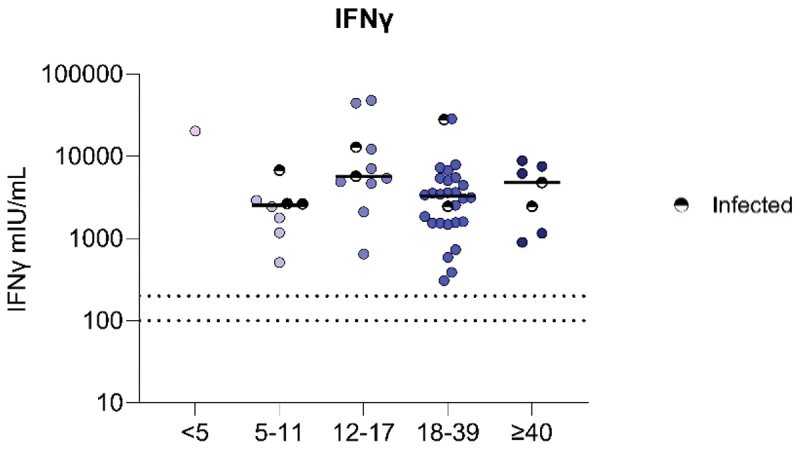
Comparable IFNγ production by SARS-CoV-2-specific T cells in DS over age after mRNA primary vaccination. IFNγ (mIu/ml) production by SARS-CoV-2-specific T cells measured by IGRA. An IFNγ concentration between 100–200 mIU/mL was considered a borderline vaccine reaction, and >200 mIU/mL a positive reaction, indicated by the dashed lines. Significance was determined using Kruskal-Wallis test with Dunn’s multiple comparisons test. The single participant <5 was not taken along for calculating significance. The median is shown as a black line. Previous infected individuals were annotated as infected based on criteria as described in Figure 1. DS < 5 N = 1, DS 5–11 N = 8, DS 12–17 N = 11, DS 18–39 N = 28, DS ≥ 40 N = 7.

To summarize, we found a decreased SI and percentage of re-activated SARS-CoV-2-specific CD4^+^ T cells after primary mRNA vaccination in children with DS aged 5–11 y compared to adolescents aged 12–17 y. Nonetheless, the functional re-activated T cell response was not impaired in children compared to older individuals with DS. This is in line with our previous findings that primary mRNA vaccination induced lower percentages of SARS-CoV-2-specific CD4^+^ T cells that became activated after S-peptide stimulation in adults with DS compared to HC, but did not affect the functional recall response based on IFNγ production.^26^ Besides a lower re-activation of SARS-CoV-2-specific CD4^+^ T cells which suggests that vaccination may not have induced a good T-cell response in children with DS, we previously observed lower antibody titers in children with DS compared to adolescents with DS after primary vaccination.^25^ Since CD4^+^ T cells play an essential role in the activation of B cells in order to produce antibodies,^36^ the potential lower induction of SARS-CoV-2-specific CD4^+^ T cells suggested by the observation of lower SI and percentages of re-activated AIM^+^ CD4^+^ T cells in children with DS, might contribute to the decreased antibody response in children younger than 12 y of age. Future research should focus on the interplay between the humoral and cellular response, as T follicular helper cells, which are essential in providing B cell help during the germinal center reaction,^37^ have been found to be decreased in children with DS compared to HC.^38^

Age plays an important role in the ability to raise an effective vaccine-induced immune response. Neonates, young infants and elderly generally have diminished humoral and cellular vaccine responses compared to adults,^39–41^ whereas children in the school-going age often show robust immune responses to vaccination and infection.^42,43^ The observed increased activation after αCD3/αCD28 dynabead stimulation in children with DS aged 5–11 y compared to adults is in line with this robust immune response. Together, this indicates that age is unlikely to explain the decreased re-activation of SARS-CoV-2–specific CD4^+^ T cells observed in children with DS. Instead, a more plausible explanation may be the lower mRNA vaccine dose administered to children aged 5–11 y compared with adolescents and adults, which could have resulted in insufficient antigenic stimulation to elicit a robust SARS-CoV-2–specific T-cell response in this group. We did not take along the N = 1 child <5 y of age for analysis, but the participant visually showed a good response, which might be linked to the three dose primary vaccine regimen. A limitation of this study is the limited number of participants we were able to include. Especially in the younger age group of 5–11 y, our results are based on N = 8 children, of which 3 children also had previous exposure to SARS-CoV-2, making it harder to compare the response to uninfected adults. Additionally, we were not able to include a HC group for the children, because they often did not get vaccinated due to a mainly mild disease course after a SARS-CoV-2 infection^44–46^ and children below 12 y of age were only advised to get vaccinated in case of increased medical risk.^29,30^ Despite not being able to compare our results obtained for children with DS to matched pediatric HC, these data suggest that the vaccine-induced T-cell response is limited in children with DS aged 5–11 y compared to children with DS aged 12–17 y. A similar pattern was shown before for the antibody titers.^25^ Therefore further investigation is necessary to determine the underlying mechanisms in children with DS with the aim to optimize prevention strategies such as vaccinations for this vulnerable population. Others have found that the magnitude of the CD4^+^ and CD8^+^ T-cell response is lower in healthy children compared to adults after a SARS-CoV-2 infection,^47^ with often undetectable CD4^+^ T-cell responses in children <4 y.^48^ They suggested that this might contribute to a milder disease course in children upon infection, however in case of vaccination, decreased cellular activation is not the outcome to strive for. The lower vaccine dose of 10 µg in children aged 5–11 y was chosen, because it caused less side effects in healthy pediatric individuals and similar immunogenicity of neutralizing antibodies compared to the 30 µg dose given to 16–25-y-old healthy individuals.^49^ Akhtar et al. observed no difference in the functional T-cell response in uninfected healthy children aged 5–10 y (N = 7) and adults (N = 11) after two doses of Pfizer, based on SARS-CoV-2-specific T cells producing IFNγ, Granzyme B or perforin.^50^ This suggests that primary vaccination with the Pfizer mRNA vaccine with a dosage of 10 µg induces an effective humoral and cellular immune response in healthy children aged 5–11 y.

To the best of our knowledge, we are the first to evaluate the cellular SARS-CoV-2 vaccine-induced immune response in children with DS. Considering the airway abnormalities, low muscle tone and the generally impaired immune system in DS,^5,6,9–12,51^ the observed reduction in re-activation of CD4^+^ T cells in children with DS aged 5–11 y compared to DS adolescents after primary vaccination, together with previously reported lower SARS-CoV-2-specific antibody titers in this age group,^25^ suggest that children with DS aged 5–11 y may benefit from receiving a vaccine dose similar to that administered to adolescents and adults with DS. This contrasts with the clinical trial data in healthy children,^49^ underscoring that immune responses in children with DS cannot be directly extrapolated from healthy pediatric populations. Future research should focus on the (possible) protective effect of a higher dosage or booster for children with DS. Given that children with DS are at increased risk of severe COVID-19 illness compared with healthy children,^16–18^ an effective vaccine-induced immune response is pertinent to ensure optimal protection. Collectively, our results emphasize the need for tailored vaccination strategies and highlight the importance of specifically evaluating vaccine-induced immune responses in immunocompromised pediatric populations such as children with DS.

## Supplementary Material

26004016_Hensen_T_cells_Children_supplementals_clean.docx

## Data Availability

All data are available upon request from the corresponding author.
