# Supplementary material for: T-cell responses to primary SARS-CoV-2 vaccination in Down syndrome – From childhood to adulthood
Source: Hum Vaccin Immunother. 2026 Jun 1;22(1):2670839. doi: 10.1080/21645515.2026.2670839 (PMC13228946; doi:10.1080/21645515.2026.2670839)
Supplement: 26004016_Hensen_T_cells_Children_supplementals_clean.docx [file KHVI_A_2670839_SM0219.docx]

**Supplemental material**

**A.**

**
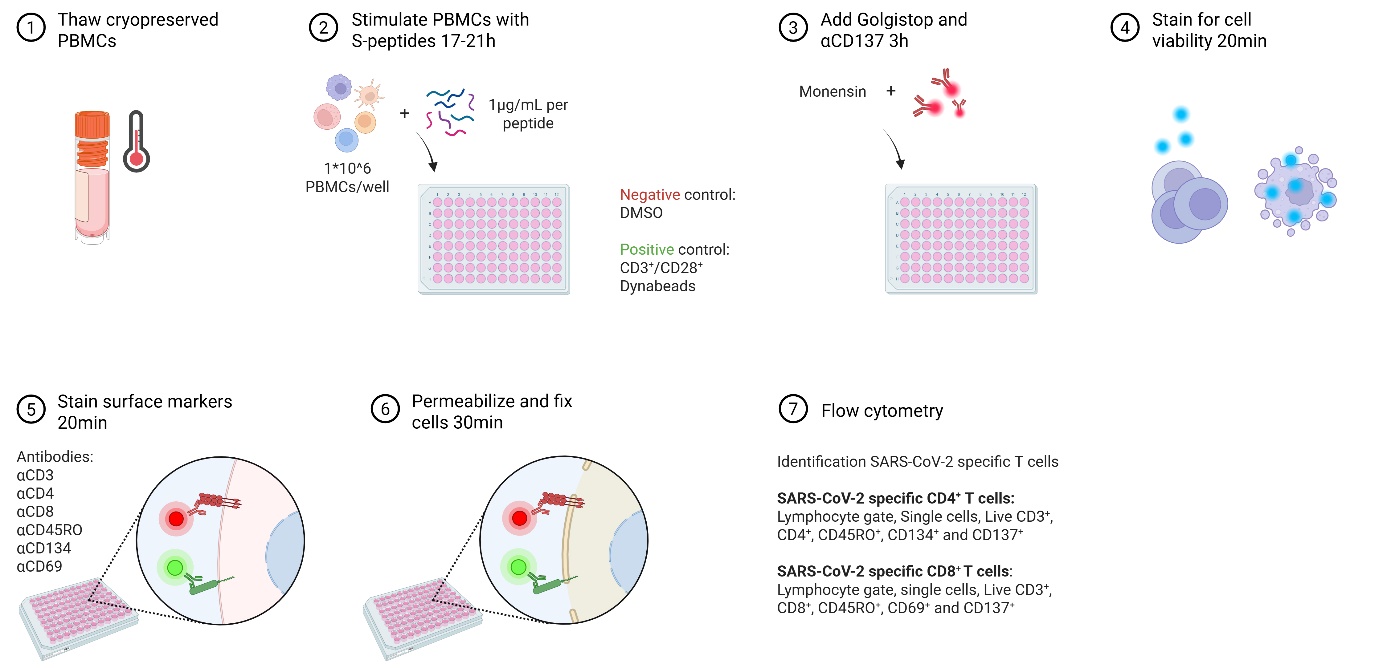
**

**B.**

**
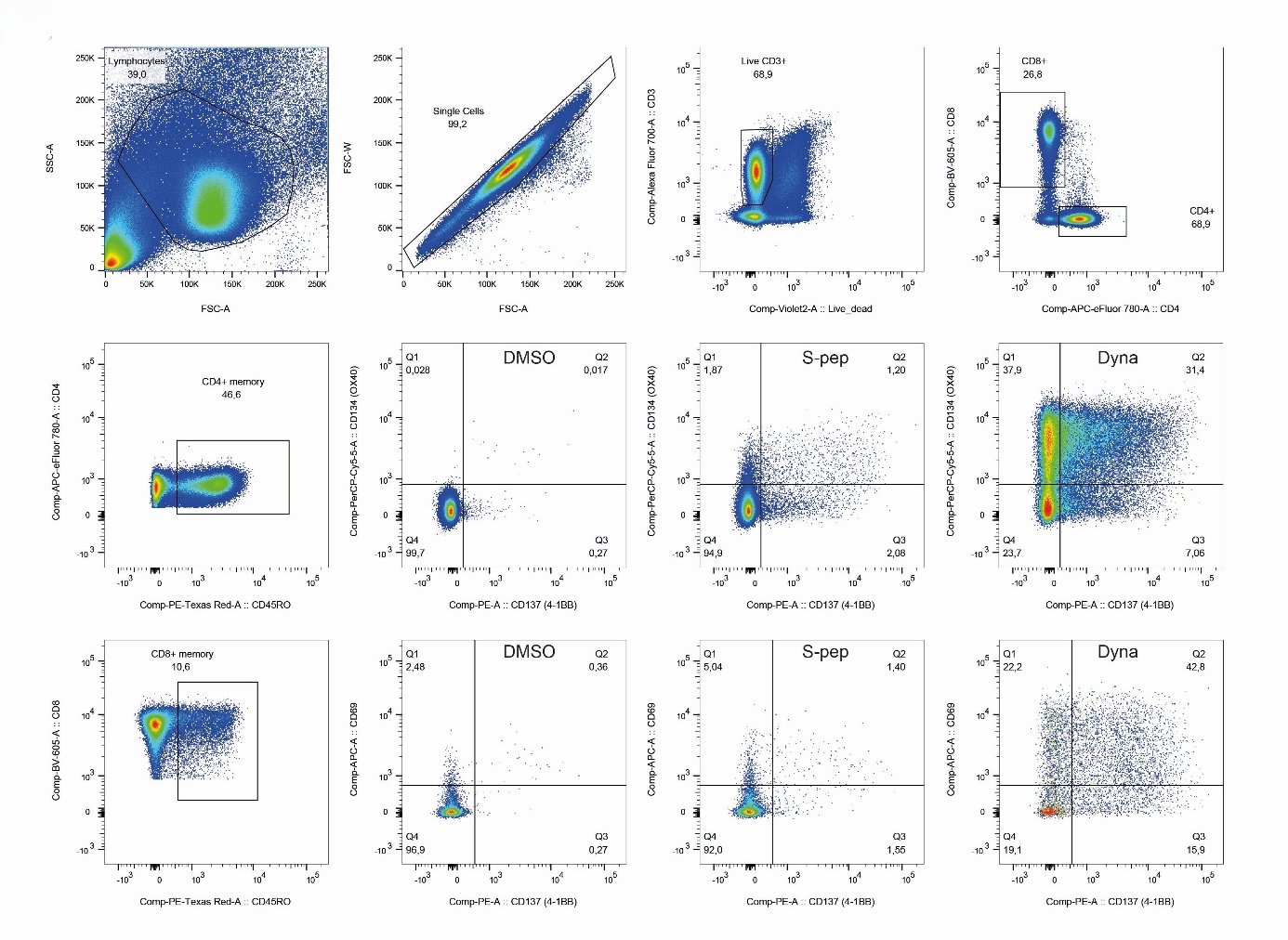
**

**Supplemental Figure 1: Activation induced marker (AIM) assay protocol and gating strategy for T cells after stimulation with DMSO, S-peptides or Dynabeads.** Figures are adapted from original paper^26^. **A.** General overview of AIM assay protocol. 1) Cryopreserved cells were thawed in 15 mL RPMI1640 completed with 2% fetal calf serum, 100 U/ml penicillin, 100 μg/ml streptomycin, and 2 mM glutamine and washed twice. PBMCs were resuspended in RPMI1640 completed with 10% human AB serum, 100 U/ml penicillin, 100 μg/ml streptomycin, and 2 mM glutamine (culture medium). 2) 1x10^6 thawed PBMCs were stimulated for a total of 20-24 hours at 37°C, 5% CO2 with a SARS-CoV-2 spike peptide pool (1 µg/mL per peptide) in 200 µL culture medium in 96-well U bottom plate. Cells were stimulated with an equimolar amount of DMSO (negative control), or with CD3/CD28 dynabeads (positive control). 3) After 17-21 hours incubation, Golgistop (1500x diluted) and CD137 antibody were added for 3 hours in culture. 4) Fc receptors were blocked with normal mouse serum for 10 min at 4°C before cells were stained with fixable viability dye for 20 min at 4°C. 5) PBMCs were surface stained with antibodies against CD3, CD4, CD8, CD45RO, CD134, and CD69 in the presence of brilliant stain buffer for 20 min at 4°C. 6) Finally, PBMCs were fixed and permeabilized with FoxP3 transcription factor staining buffer for 30 minutes at 4°C, and 7) Hereafter, PBMCs were measured with flow cytometry (BD LSR Fortessa, BD Bioscience) and data analyzed in FlowJo V10.8.1, BD Bioscience. 8) SARS-CoV-2 specific T cell were identified based on these markers after S-peptide stimulation. Created in BioRender. Hensen, L. (2026). **B.** Gating strategy of T cells after stimulation with DMSO, spike peptides (S-pep) or dynabeads (Dyna). Single cells were gated for live CD3^+^ T cells and subdivided into CD3^+^CD4^+^ T helper cells and CD3^+^CD8^+^ T-cytotoxic cells. Memory T cells were identified by gating CD45RO^+^ within CD4^+^ or CD8^+^ subsets. SARS-CoV-2-specific memory T cells (AIM^+^ T cells) were defined as CD134^+^CD137^+^ for T helper cells and CD69^+^CD137^+^ for cytotoxic T cells. The DMSO-stimulated sample was used to set the cutoff gate for activation markers.

**
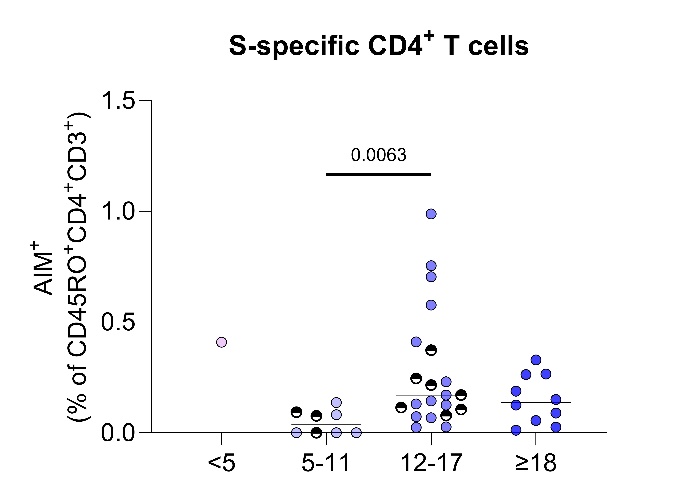
**
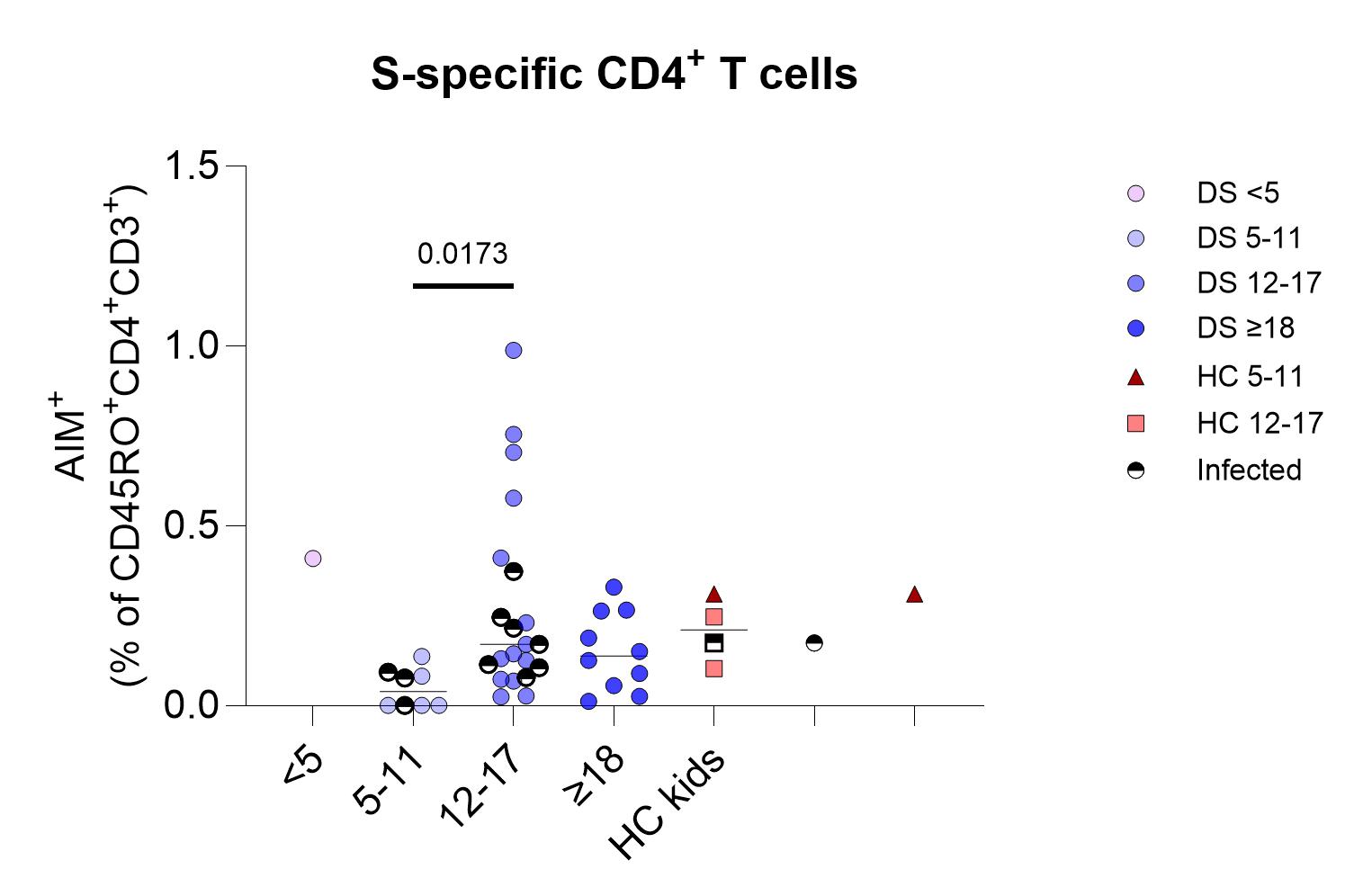


**Supplemental Figure 2: Lower percentage of SARS-CoV-2-specific CD4^+^ T cells in children with DS between 5-11 years of age after primary mRNA vaccination.** Percentage of AIM^+^ cells within the CD4^+^CD45RO^+^ subset after background subtraction. AIM^+^CD4^+^ cells are defined as CD134^+^CD137^+^. Significance was determined using Kruskal-Wallis test with Dunn’s multiple comparisons test. The single participant <5 was not taken along for calculating significance. The median is shown as a black line. Previous infected individuals were annotated as infected based on criteria as described in Figure 1. DS<5 N=1, DS 5-11 N=8, DS 12-17 N=21, DS≥18 N=10.

**
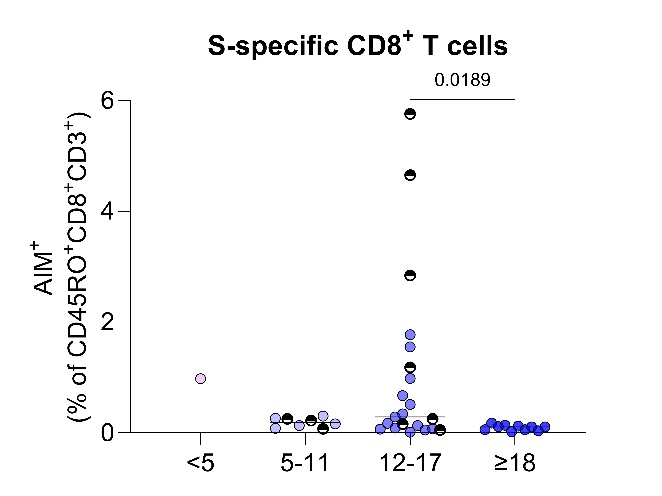

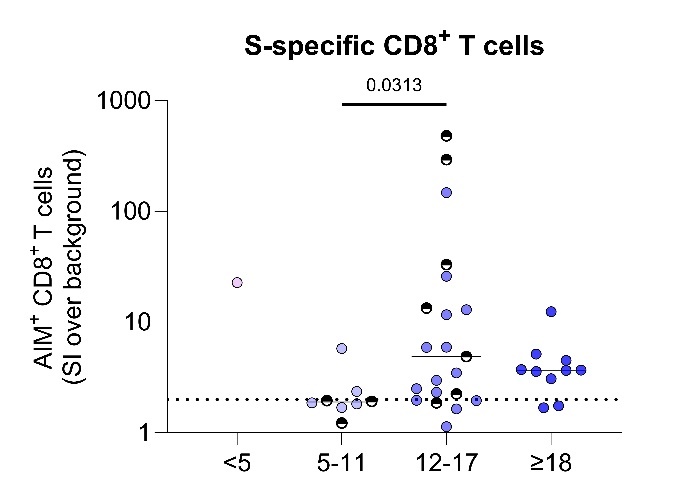
**

B

A


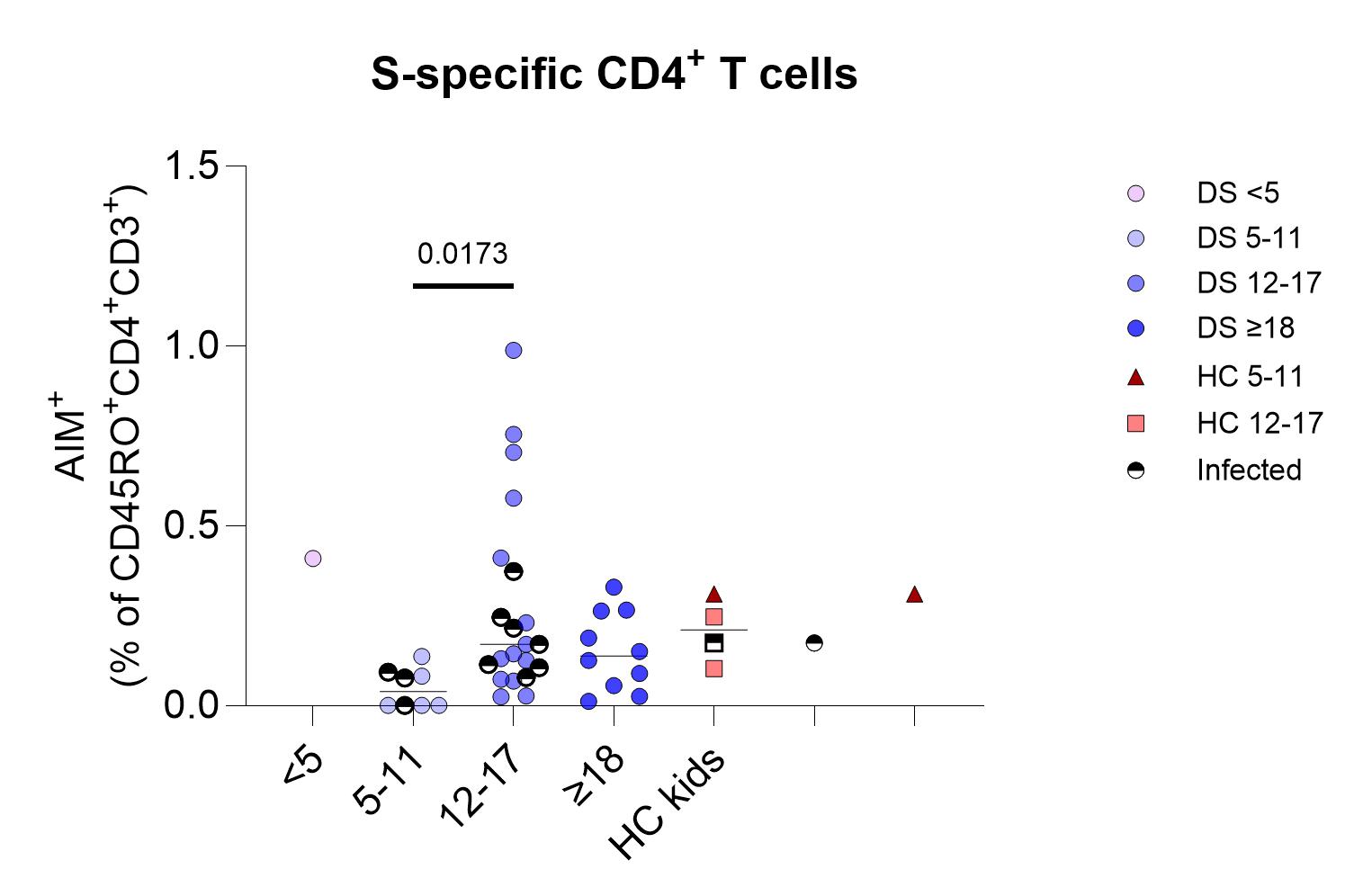


**Supplemental Figure 3: Stimulation index and percentage of SARS-CoV-2-specific CD8^+^ T cells in DS over age after primary mRNA vaccination. (A)** Stimulation index (SI) of AIM^+^CD8^+^ T cells, calculated by dividing specific activation over background activation. AIM^+^CD8^+^ cells are defined as CD69^+^CD137^+^. An SI of 2 or higher is considered a positive T-cell response (dashed line). **(B)** Percentage of AIM^+^ cells within the CD8^+^CD45RO^+^ subset after background subtraction. Significance in **(A-B)** was determined using Kruskal-Wallis test with Dunn’s multiple comparisons test. The single participant <5 was not taken along for calculating significance. The median is shown as a black line. Previous infected individuals were annotated as infected based on criteria as described in Figure 1. DS<5 N=1, DS 5-11 N=8, DS 12-17 N=21, DS≥18 N=10.

**Supplemental Table 1: Overview of materials.** Table adapted from original^26^.

| **Antibodies** | | | | | | | |
| --- | --- | --- | --- | --- | --- | --- | --- |
| *Target* | *Label* | *Vendor* | *Clone* | *Catalog#* | *Usage* | *RRID* |  |
| Fixable Viability Dye | eFluor506 | eBioscience | N.A. | 65-0866-14 | 1000x | N.A. |  |
| CD3 | AF700 | Biolegend | UCHT1 | 300424 | 50x | AB_493741 |  |
| CD4 | APC-eF780 | eBioscience | RPA-T4 | 47-0049-42 | 200x | AB_1272044 |  |
| CD8 | BV605 | Biolegend | SK1 | 344742 | 50x | AB_2566513 |  |
| CCR7 | APC | Biolegend | G043H7 | 353214 | 40x | AB_10917387 |  |
| CD45RO | ECD | Beckman Coulter | UCHL1 | B49192 | 25x | AB_3073671 |  |
| CD31 | FITC | BD Biosciences | WM59 | 555445 | 50x | AB_395838 |  |
| CD19 | APC-eF780 | eBioscience | HIB19 | 47-0199-42 | 20x | AB_1582230 |  |
| CD137 | PE | BD Biosciences | 4B4-1 | 555956 | 50x | AB_396252 |  |
| CD134 | PerCP-Cy5.5 | Biolegend | Ber-ACT35 | 350010 | 25x | AB_10901161 |  |
| CD69 | APC | Biolegend | FN50 | 310910 | 100x | AB_314845 |  |
| **Reagents** | | | | |  | | |
| *Name* | | *Vendor* | | *Catalog#* | *Usage* | |  |
| RPMI 1640 medium | | Gibco | | 12017599 | N.A. | |  |
| Normal mouse serum | | Bioconnect | | 88-NM35 | 25x | |  |
| FcR blocking reagent human | | Miltenyi Biotec | | 130-059-901 | 2% | |  |
| FoxP3 transcription factor staining buffer set | | eBioscience | | 00-5523-00 | According to manufacturer | |  |
| PepMix SARS-CoV-2 (Spike Glycoprotein) | | JPT | | PM-WCPV-S-1 | 1 µg/mL per peptide | |  |
| DMSO | | Sigma-Aldrich | | D5879 | Equimolar as peptide pool | |  |
| Dynabeads Human T-activator CD3/CD8 | | Invitrogen | | 10587973 | 1 bead per 5 PBMCs | |  |
| GolgiStop | | BD Bioscience | | 554724 | 1500x | |  |
| Brilliant Stain buffer | | BD Bioscience | | 563794 | 8% | |  |
| Quan-T-Cell SARS-CoV-2 | | EUROIMMUN | | ET 2606-3003 | According to manufacturer | |  |
| Quan-T-Cell ELISA | | EUROIMMUN | | EQ 6841-9601 | According to manufacturer | |  |
| **Devices and software** | | | | | | | |
| *Name* | | *Source* | | | | |  |
| Clariostar plate reader | | BMG LABTECH | | | | |  |
| BD LSR Fortessa (4-laser) flow cytometer | | BD Bioscience | | | | |  |
| FlowJo V10.8.1 | | BD Bioscience | | | | |  |
| Prism 10.1.2 | | Graphpad | | | | |  |
| Abbott Alinity hq analyzer | | Abbott | | | | |  |

**Supplemental Table 2:** **Timing of infection for each participant of the AIM assay.** Previous infection with SARS-CoV-2 was determined based on a positive anti-S IgG concentration (>10.08 BAU/ml) at baseline (T1) or a positive anti-N IgG concentration (>14.3 BAU/ml) at baseline, at T2, approximately 28 days after the first vaccination, or T3, approximately 28 days after second vaccination^51^.

| **Age (years)** | **Positive anti-N and/or anti-S at T1 titers (timepoint)** |
| --- | --- |
| 9 | T1, T3 |
| 9 | T1 |
| 11 | T3; T1 & T2 unknown |
| 12 | T3; T1 & T2 unknown |
| 12 | T1, T3; T2 unknown |
| 13 | T3; T1 & T2 unknown |
| 13 | T1 |
| 14 | T3; T1 & T2 unknown |
| 15 | T3; T1 & T2 unknown |
| 16 | T1, T2, T3 |

**Supplemental Table 3:** **Timing of infection for each participant of the IGRA.** Previous infection with SARS-CoV-2 was determined based on a positive anti-S IgG concentration (>10.08 BAU/ml) at baseline (T1) or a positive anti-N IgG concentration (>14.3 BAU/ml) at baseline, at T2, approximately 28 days after the first vaccination, or T3, approximately 28 days after second vaccination^51^.

| **Age (years)** | **Positive anti-N and/or anti-S at T1 titers (timepoint)** |
| --- | --- |
| 9 | T1, T3 |
| 9 | T1 |
| 11 | T3; T1 & T2 unknown |
| 12 | T1, T3; T2 unknown |
| 16 | T1, T2, T3 |
| 26 | T2, T3 |
| 27 | T3 |
| 57 | T1 |
| 57 | T3; T1 & T2 unknown |
